# Endogenous Oxytocin Levels in Autism—A Meta-Analysis

**DOI:** 10.3390/brainsci11111545

**Published:** 2021-11-21

**Authors:** Matthijs Moerkerke, Mathieu Peeters, Lyssa de Vries, Nicky Daniels, Jean Steyaert, Kaat Alaerts, Bart Boets

**Affiliations:** 1 Center for Developmental Psychiatry, Department of Neurosciences, KU Leuven, 3000 Leuven, Belgium; mathieu.peeters@student.kuleuven.be (M.P.); lyssa.devries@kuleuven.be (L.d.V.); jean.steyaert@kuleuven.be (J.S.); bart.boets@kuleuven.be (B.B.); 2 Leuven Autism Research (LAuRes), KU Leuven, 3000 Leuven, Belgium; nicky.daniels@kuleuven.be (N.D.); kaat.alaerts@kuleuven.be (K.A.); 3 Research Group for Neurorehabilitation, Department of Rehabilitation Sciences, KU Leuven, 3000 Leuven, Belgium

**Keywords:** endogenous oxytocin, autism spectrum disorder, biological marker

## Abstract

Oxytocin (OT) circuitry plays a major role in the mediation of prosocial behavior. Individuals with autism spectrum disorder (ASD) are characterized by impairments in social interaction and communication and have been suggested to display deficiencies in central OT mechanisms. The current preregistered meta-analysis evaluated potential group differences in endogenous OT levels between individuals with ASD and neurotypical (NT) controls. We included 18 studies comprising a total of 1422 participants. We found that endogenous OT levels are lower in children with ASD as compared to NT controls (n = 1123; g = −0.60; *p* = 0.006), but this effect seems to disappear in adolescent (n = 152; g = −0.20; *p* = 0.53) and adult populations (n = 147; g = 0.27; *p* = 0.45). Secondly, while no significant subgroup differences were found in regard to sex, the group difference in OT levels of individuals with versus without ASD seems to be only present in the studies with male participants (n = 814; g = −0.44; *p* = 0.08) and not female participants (n = 192; g = 0.11; *p* = 0.47). More research that employs more homogeneous methods is necessary to investigate potential developmental changes in endogenous OT levels, both in typical and atypical development, and to explore the possible use of OT level measurement as a diagnostic marker of ASD.

## 1. Introduction

Autism spectrum disorder (ASD) is a pervasive neurodevelopmental disorder characterized by deficits in reciprocal social communication and interaction, and by the presence of restricted, stereotyped and repetitive interests and behaviors [1]. This early onset developmental disorder has a substantial genetic predisposition and affects mostly boys [2]. The multifactorial etiology and underlying neurobiological mechanisms responsible for the heterogeneous clinical presentation of ASD are not yet fully understood, limiting diagnosis of ASD to rely solely on developmental history, behavioral and cognitive assessment. While current diagnostic approaches do not involve any biomarkers, recent evidence suggests that core symptoms of social deficits featured in ASD might be associated with oxytocin (OT) dysfunction in the central nervous system [3,4,5,6,7,8,9,10].

OT is a hypothalamic nonapeptide with a wide variety of bodily functions. It is produced in the neurons of the paraventricular and supraoptic nuclei of the hypothalamus. These nuclei project to the posterior pituitary gland, where OT is released into the systemic circulation. In peripheral tissues, OT acts as a key mediator in labor induction and lactation [11]. In the central nervous system, OT is an important modulator of socio-cognitive functions and complex social behaviors (e.g., empathy, emotion recognition, attachment development and affiliative behaviors) through action in different brain regions (e.g., amygdala) [12,13]. Against this background, researchers have examined potential prosocial effects of intranasal OT administration, both in healthy individuals [14] and in clinical populations [15]. In their systematic review of OT effects in healthy individuals, Mierop et al. (2020) described large heterogeneity, limited and unsuccessful replication studies and low statistical power, thus all pointing to a restricted possibility to disentangle true from false OT effects [14]. With respect to ASD, clinical trials generally report that intranasal OT has the potential to improve social impairment, or at least in particular individuals [16,17,18,19]. Parker et al. (2017), for instance, found that the therapeutic effect of intranasal OT administration was strongest in those ASD children showing the lowest endogenous OT levels pre-treatment [18]. Similarly, Alaerts et al. (2021) showed improvements in social behavior in adults with ASD after four weeks of intranasal OT administration, and these social improvements were accompanied by elevated endogenous OT levels until one month later [20]. These findings indicate the importance of endogenous OT levels as a potential indicator of OT therapy outcome.

Previous studies have shown correlations between individual differences in endogenous OT levels and social deficits, suggesting a potential role of endogenous OT in the pathogenesis of social impairments that characterize ASD [21]. However, an earlier meta-analysis by Rutigliano and colleagues (2016) that integrated findings on six adult studies reported no significant group differences in OT concentrations comparing adults with ASD versus neurotypical (NT) controls [22]. Though, previous evidence suggested a developmental trend in the association of endogenous OT levels and socio-emotional functioning, with a more pronounced association in younger age groups [23]. Therefore, it is critical to consider a wider age range for the group comparisons. Accordingly, the current systematic review and meta-analysis aims at investigating whether there are differences in endogenous OT concentrations in individuals with ASD compared to matched NT controls across all ages.

## 2. Materials and Methods

A protocol describing the rationale and methods of this meta-analysis was registered on PROSPERO (registration number: CRD42021231207) on 14 February 2021. It is available at https://www.crd.york.ac.uk/prospero/display_record.php?ID=CRD42021231207 (accessed on 18 November 2021).

Studies were eligible for inclusion if a direct comparison of endogenous OT concentrations was reported between a group of human participants with a formal ASD diagnosis versus an NT control group. Reports of (group differences in) endogenous OT levels in blood serum, blood plasma, urine or saliva were required as an outcome. Interventional studies were included if baseline pre-intervention OT levels were assessed and compared between the ASD and NT groups. Participants of any age, sex and ethnicity were considered. No restrictions were imposed regarding methods of tissue sample extraction, sample measurement or statistical analysis of the OT levels. Concerning the target population, all participants received a diagnosis of ASD according to the Diagnostic and Statistical Manual of Mental Disorders, fourth (DSM-IV), fourth text reviewed (DSM-IV-TR) or fifth edition (DSM-5) prior to the study and had no physical or severe psychiatric comorbidities (e.g., schizophrenia; bipolar disorder). No exclusion criteria were defined based on disorder subtype (i.e., Asperger, autistic disorder or PDD-NOS) or symptom severity. In terms of the NT control population, all included studies reported that the NT participants displayed no physical disease, neurological or mental disorder. We only included between-group designs, thereby excluding all studies involving single group designs, case studies, reviews, meta-analyses and animal studies. Only papers published in English were considered. No restrictions were imposed regarding publication year.

An electronic search strategy was constructed according to PRISMA guidelines, aimed at identifying all studies comparing endogenous OT concentrations between an ASD group and an NT control group. Electronic bibliographic databases Pubmed and Embase were searched up to 1 December 2020. The following string of key terms was used: (oxytocin OR OT) AND (autism OR autism spectrum disorder OR ASD OR autistic). Search terms were applied to title, abstract and keywords. In the process of study selection, the title and abstract of all studies that resulted from our systematic literature search were screened for eligibility. All duplicates and studies meeting one or more exclusion criteria were excluded. In addition, studies without available full text were excluded. Articles resulting from the screening process underwent full text analysis to evaluate if studies were to be included based on the previously determined eligibility criteria. In addition to the predefined electronic search strategy, reference lists of the selected articles were inspected for further empirical studies that may meet inclusion criteria. 

Prior to statistical analyses, all included studies were reviewed and the following information was extracted: (i) participant characterization (sample sizes, age, sex distribution, diagnostic criteria), (ii) specimen type, method of sample collection and measurement, OT concentration, (iii) effect sizes of group comparisons and/or descriptive statistics allowing the calculation of standardized effect sizes. Studies qualified for inclusion in the formal meta-analysis if the means and standard deviations for both groups were provided or could be retrieved. If the necessary study data were insufficient, the corresponding author of the study was contacted by e-mail to request provision of the missing data. A reminder request was sent within 14 days if the authors did not respond to the first e-mail. For two studies that did not report the necessary descriptive statistics in texts or tables, but only graphically represented in a figure, an online plot digitizer was used to estimate the mean and standard deviation of the concentrations displayed in the figures [24]. The reliability of this digital tool was demonstrated in an earlier meta-analysis on the correlation of central and peripheral OT [25], and was additionally validated and confirmed based on the current data (i.e., by comparing the visually estimated and the reported descriptive statistics). Quantitative analyses were conducted using RevMan 5.4.1 software [26]. Overall effect sizes were calculated based on OT mean and standard deviations of ASD and NT group, weighted by sample or subsample size using a random effects model to adjust for standard errors. Effect sizes were expressed as the standardized mean difference measure Hedges’ g. An effect size of 0.2−0.3 is often considered as a small effect, 0.5 as a medium effect and 0.8 or more as a large effect [27].

We used the QUADAS-2 tool [28], which is designed specifically to evaluate the quality of diagnostic accuracy studies, to assess the risk of bias in our included articles. The QUADAS-2 tool assesses study quality at four levels, i.e., patient selection, index test, reference standard and flow/timing. Patient selection was evaluated based on the type of participant sampling applied in the study (consecutive, random), amongst other parameters. The evaluation of the conduct and interpretation of the index test, OT dosing, involved whether this was done without knowledge of the reference standard, for example. The reference standard, i.e., the diagnostic instrument used to classify the ASD group, was also assessed. The conduct of both index and reference test was assessed based on their timing and flow, for example the time between both and the order they were performed in. Finally, the participant flow was evaluated to assess whether all studied participants were included in the analyses because patients lost to follow-up can differ systematically from those who remained. Additionally, a judgment of applicability was made on each level to assess whether the study matches the research question.

## 3. Results

### 3.1. Study Selection and Characteristics 

**Figure 1:**
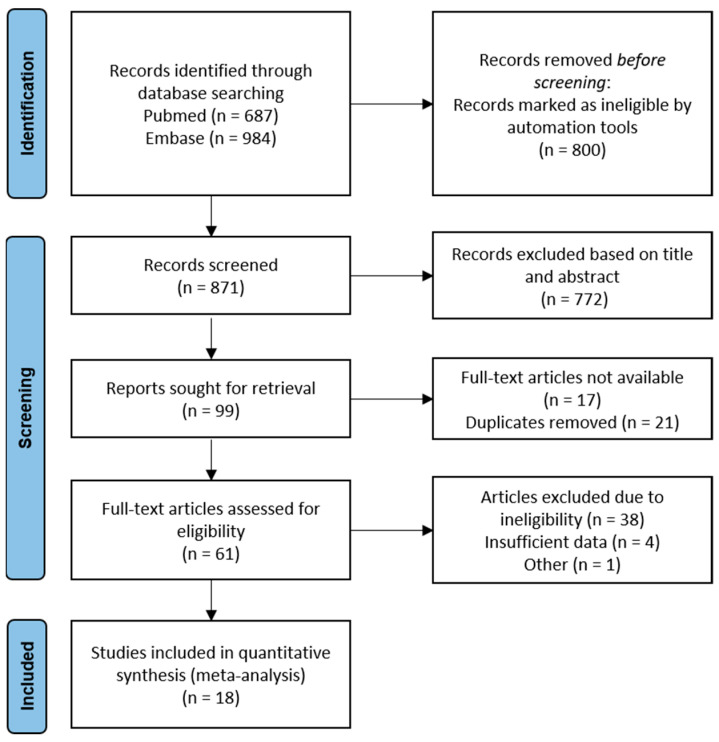
Schematic flowchart of the search method.

The literature search yielded a total of 687 and 984 articles in Pubmed and Embase, respectively. The flowchart presented in Figure 1 summarizes the number of studies that were screened, evaluated for eligibility and included in the meta-analysis. From the pool of 23 studies eligible for quantitative analysis, four studies were excluded due to insufficient data. Considering that two of the included studies reported identical data on the same participant group, only the chronologically first reported was included in the analysis [7,29]. 

An overview of the included studies and participant characteristics is provided in Table 1. Data extracted and represented in Table 1 contain author and year of publication, total group size, number of ASD and NT participants per group, sex, age, tissue sample type, method of OT level measurement, OT concentration per group and unit of measurement. A total of 18 studies comprising 1422 participants (699 ASD patients and 723 controls) were included in the final analyses. 

To further explore what underlying mechanisms might be driving the general results, more detailed subgroup analyses were performed by age, by sex and by tissue sample type. Pertaining to age, 13 studies involved comparisons of groups of children aged between 2 to 12 years [3,4,5,6,7,9,10,30,31,32,33,34,35]. Two studied adolescent groups aged between 11 to 16 years [8,36]. Three investigated groups of adults [30,31,32]. Pertaining to sex, eight studies reported on exclusively male populations [4,5,7,8,32,35,37,38] and one study adopted an exclusively female sample [32]. For the studies involving a mixed population, four studies reported separate outcomes of OT level measurement for male and female subgroups and were entered accordingly in the subgroup analyses [33,34,35,36]. With regard to tissue sample type, twelve studies investigated blood plasma [3,4,5,7,9,10,30,32,34,35,36,37]. Six studies took saliva samples for OT level measurement [6,8,31,33,38,39].

### 3.2. Qualitative Risk of Bias Analysis of Included Studies

The risk of bias evaluation for each individual study is shown in Table 2. None of the studies show a ‘low’ risk of bias on all four levels, most often the total risk of bias remains ‘unclear’ rather than ‘high’. Applicability concerns are low. Whilst being aware of the moderate quality of the reviewed studies, we nevertheless decided to include all studies in our analyses because of the limited amount of available studies.

### 3.3. Meta-Analysis of Peripheral OT Levels in ASD vs. NT Controls

**Figure 2:**
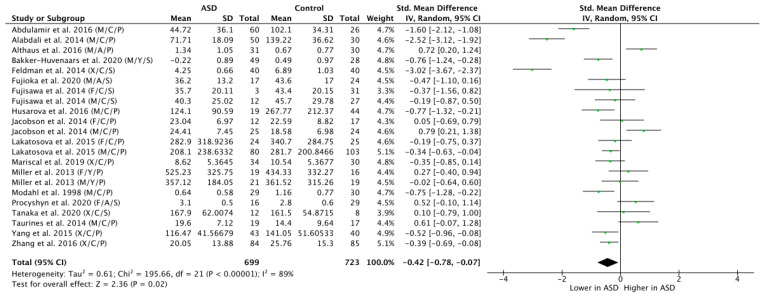
Meta-analysis of peripheral OT levels in ASD vs. NT. M = male, F = female, X = mixed sexes; C = children, Y = youths/adolescents, A = adults; P = plasma, S = saliva.

Across all the 18 studies, meta-regression of differences in endogenous OT between ASD and NT populations resulted in a mean standardized difference of g = −0.42, which was significant (n = 1422, g = −0.42, Z = 2.36, *p* = 0.02, CI = (−0.78, −0.07)) and indicates that OT levels are generally lower in participants with ASD as compared to NT controls (see Figure 2). Yet, the effect was highly heterogeneous (Tau^2^ = 0.61; Chi^2^ = 195.66, df = 21 (*p* < 0.00001); I^2^ = 89%), thereby motivating a further subgroup analysis.

### 3.4. Subgroup Analysis by Age 

**Figure 3:**
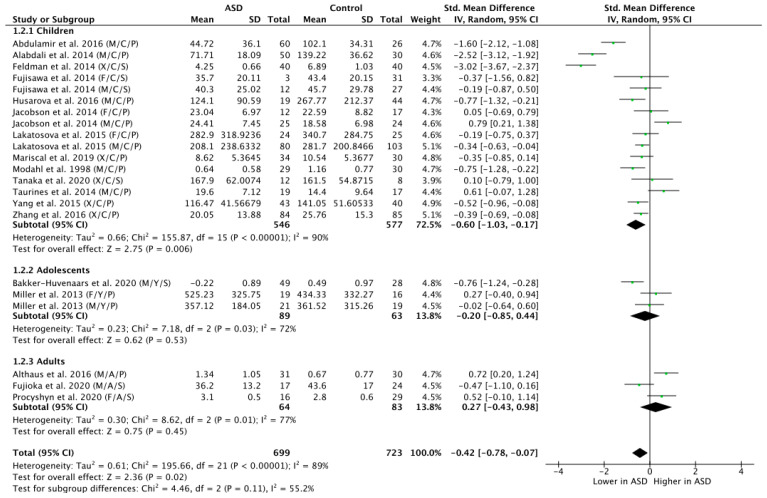
Subgroup analysis by age. M = male, F = female, X = mixed sexes; C = children, Y = youths/adolescents, A = adults; P = plasma, S = saliva.

The subgroup analysis by age revealed that ASD children did display significantly lower OT levels compared to NT children (n = 1123; g = −0.60; Z = 2.75; *p* = 0.006; CI = (−1.03, −0.17)) (see Figure 3). However, there were no significant group differences in the OT levels for the adolescent populations (n = 152; g = −0.20; Z = 0.62; *p* = 0.53; CI = (−0.85, −0.44)), nor for the adult populations (n = 147; g = 0.27; Z = 0.75; *p* = 0.45; CI = (−0.43, −0.98)).

### 3.5. Subgroup Analysis by Sex 

**Figure 4:**
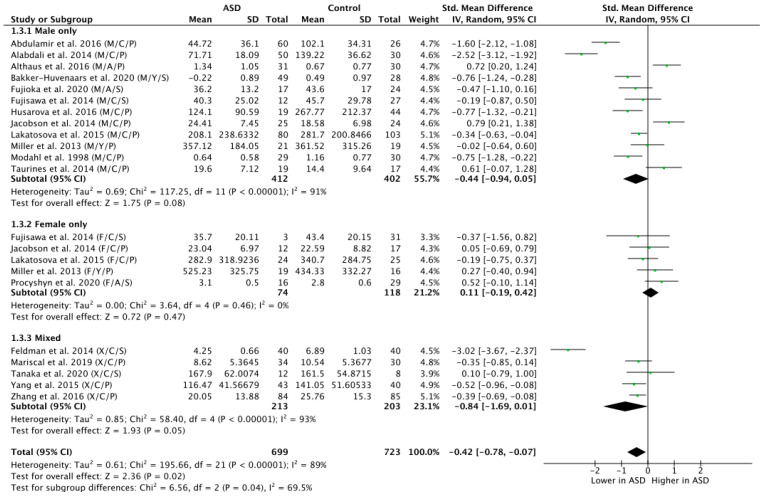
Subgroup analysis by sex. M = male, F = female, X = mixed sexes; C = children, Y = youths/adolescents, A = adults; P = plasma, S = saliva.

To explore the potential influence of sex on OT levels in ASD versus NT, a subgroup analysis by sex was carried out by grouping participants on the basis of sex and contrasting all exclusively male, female and mixed (i.e., papers where disentanglement was impossible) groups (Figure 4). This analysis revealed a trend-level effect of lower OT levels in the ASD group in the male population (n = 814; g = −0.44; Z = 1.75; *p* = 0.08; CI = (−0.94, 0.05)) but no significant effect in the female population (n = 192; g = 0.11; Z = 0.72; *p* = 0.47; CI = (−0.19, 0.42)). The mixed group, however, showed significantly lower OT levels in ASD versus NT (n = 416; g = −0.84; Z = 1.93; *p* = 0.05; CI = (−1.69, 0.01)).

### 3.6. Subgroup Analysis by Tissue Sample 

**Figure 5:**
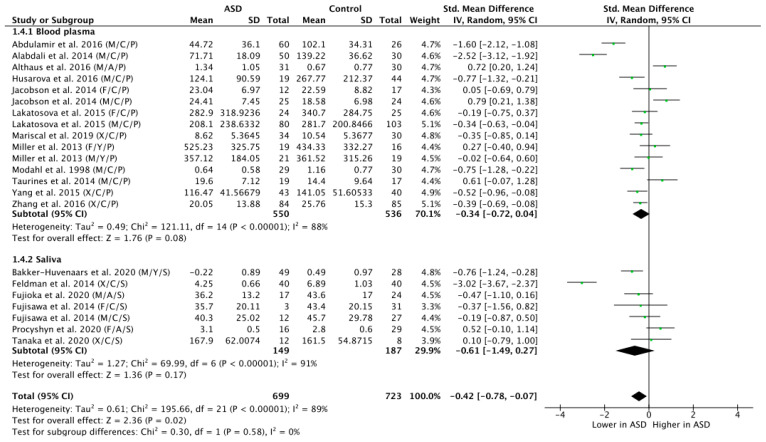
Subgroup analysis by tissue sample. M = male, F = female, X = mixed sexes; C = children, Y = youths/adolescents, A = adults; P = plasma, S = saliva.

A subgroup analysis by tissue sample (blood plasma or saliva) showed a trend-level group difference in OT levels of ASD versus NT participants as assessed on the basis of blood plasma concentrations (n = 1086; g = −0.34; Z = 1.76; *p* = 0.08; CI = (−0.72, 0.04)) (Figure 5). While the effect size calculated on the basis of saliva as specimen for OT group comparison was high (g = −0.61), it did not reach significance, due to the larger variability and the smaller number of included participants (n = 336; g = −0.61; Z = 1.36; *p* = 0.17; CI = (−1.49, 0.27)). Statistically testing for subgroup differences did indeed confirm that the significant overall group effect was not modulated on the basis of tissue sample type subgroup differences (Chi^2^ = 0.30, df = 1; *p* = 0.58; I^2^ = 0%).

## 4. Discussion

There is growing evidence that OT circuitry plays a major role in the mediation of prosocial behavior, including empathy, emotion recognition, eye contact and attachment development [40]. Individuals with ASD are characterized by impairments in social interaction and communication, and have been suggested to display deficiencies in central OT mechanisms. The current study evaluated potential group differences in endogenous OT levels between ASD and NT controls, and considered age, sex and tissue type as potential mediators of this group difference. We included 18 studies comprising a total of 1422 participants. 

Statistical analysis of the total pool of participants showed that endogenous OT levels in ASD populations are significantly lower compared to NT controls. These results are in contradiction with an earlier systematic review and preliminary meta-analysis addressing peripheral OT in psychiatric disorders in general [22], in which no significant difference was found in plasma or salivary OT between ASD and NTs. However, the studies included in this review only comprised adult participants. To elucidate this discrepancy, we performed a subgroup analysis by age, which showed that children with ASD do have significantly lower OT values compared to typically developing controls, but this effect disappeared in the adolescent and adult groups. This developmental effect is even further specified when contrasting the child studies, based on the average age of the participants. In particular, in the group of <6-year-olds, four out of five studies found significantly lower OT levels in ASD [3,6,9,33,34]. Similarly, in the group of 6 to 9-year-olds, four out of five studies reported significantly lower OT levels in children with ASD [4,5,7,10,35]. Yet, in the group of children >9 years old, none of the three studies reported significantly lower OT in the ASD group [30,31,32], indicating either a normalization (increase) of OT levels in ASD or a reduction in OT levels in NT controls, after early childhood. Conversely, Lakatosova et al. divided their child sample into two groups according to age (younger versus older than 10 years) and found the decrease in plasma OT to be especially prominent in the older children [35]. 

On average, significantly lower OT levels are not found in adolescents with ASD, but this may be due to the limited number of studies on this age group. Note that one study [8] did demonstrate lower OT levels in adolescents with ASD [8], but the other [36] did not observe any group differences [36]. However, pertaining to the adult populations, the general absence of observations of lower endogenous OT levels in adults with ASD seems robust and coincides with the results of the meta-analysis by Rutigliano et al. [22]. Notably, one adult study even found significantly higher OT concentrations in the ASD population [37], substantiating a potential developmental effect. 

From animal studies, we know that OT levels have organizational effects on the brain (and behavior) during specific critical time-periods, such as the postnatal or peripubertal term (REF Miller and Caldwell, 2015). How human central OT levels exactly vary during development is not yet well studied. However, the age-dependent aberrancy of the OT system, as found in the current study, has also been described in two earlier studies. First, Freeman et al. (2018) [41] found a significant negative association between age and OT receptor density in the ventral pallidum (part of the reward system in the brain) in individuals with and without ASD. When inspecting these results more closely, they found an early-life peak in OT receptor density in NT children, which was absent in ASD children [41]. These authors interpreted these findings by suggesting that the lack of this early-life critical period, where this brain area becomes maximally sensitive to oxytocin binding and social reward, may impact social development and may thus result in social symptoms in ASD. Second, a recent review on oxytocin receptor (OXTR) gene DNA methylation suggested hypomethylation in children with ASD and hypermethylation in adults with ASD [42]. Tentatively, this opposite developmental pattern was interpreted as if the initial hypomethylation in children with ASD may underlie their aversive and intrusive experience of social encounters, which they gradually counter by developing a hypermethylated (hence, dampened) OXTR system. Given the current observation of lower circulating OT levels in children with ASD and the lack of an early-life peak in OT receptor density, the suggested OXTR hypomethylation in children with ASD could also be interpreted as an inefficient biological manner of coping with these OT system deficiencies.

Next, we performed a subgroup analysis per sex. This analysis yielded a marginally significantly lower OT level in males, but clearly no group difference in females. Note that the effect size in the male subgroup is considerable (g = −0.44, *p* = 0.08) and even exceeds the overall effect size across all studies (g = −0.42, *p* = 0.02), while the effect size in the females is negligible (g = 0.11, *p* = 0.47). The male group is, however, larger (n = 814) than the female group (n = 192), which could also play a part here. An additional analysis investigating also two-factor subgroup interactions of age and sex revealed (marginally) lower OT levels in boys (*p* = 0.08) and mixed boys/girls (*p* = 0.05) groups of children with ASD but not in girls with ASD (*p* = 0.53) (Figure S1). Lakatosova et al. consistently reported relatively decreased plasma OT levels in boys with ASD but not in girls with ASD [35]. ASD is a male-dominant disorder and the diagnosis is three times more prevalent in boys compared to girls according to the meta-analysis by Loomes et al. [2]. The authors attribute this mainly to the fact that boys have a more distinct and recognizable phenotype of autistic traits, whereas girls have a subtler presentation of autism characteristics and are more likely to camouflage their impairments, augmenting the risk of a late or overlooked diagnosis. As OT plays a key role in mediating social characteristics and behavior, it is possible that this phenotypical disparity between ASD males and females is driven by sex specific differences in OT dysfunctions at the neurobiological level. We know from animal studies that OT levels are indeed sexually dimorphic, and are generally much higher in females than males, which could be due to its interactions with estrogen and estrogen receptors [43,44,45,46]. Against this background, it can be hypothesized that these generally higher OT levels in females may act in a protective manner, also in females with ASD profiles. 

Obtaining central OT levels is challenging, therefore researchers acquire peripheral OT levels through blood plasma or saliva, thereby offering an accessible window on central OT circuitry. The subgroup analysis per tissue sample showed marginally lower OT levels in ASD as measured via plasma, but not via saliva. Yet, it should be noted that the effect size for saliva is considerable and even larger than plasma (g = −0.34 for plasma and −0.61 for saliva). Accordingly, the absence of a significant ASD versus NT group difference in the saliva samples largely depends on lacking statistical power due to the smaller number of participants. An active controversy regarding the measurement of OT in biological fluids concerns whether these peripheral measures are actually reliable and valid approximations for levels of OT in the central nervous system [47]. In this regard, a recent meta-analysis revealed a positive correlation between peripheral OT levels and OT levels in the central nervous system, in particular after experimental stress induction. As no correlation was observed under baseline conditions, it remains questionable to what extent peripheral OT levels may approximate central OT levels [25]. 

Against this background, one may also wonder via what mechanism reduced endogenous peripheral OT levels may impact on social functioning in children with ASD. At the peripheral level, OT has been postulated and demonstrated to exert an anxiolytic influence by regulating the cardiovascular and autonomous nervous system, and consequently reducing physiological stress reactivity and (social) anxiety [48,49]. Since OT levels are typically measured peripherally in humans, they cannot offer brain region specific information. However, from rodent studies we know that there is the widespread expression of OT receptors in distinct (human) “social” brain regions, including the amygdala and prefrontal cortex, and reward systems such as nucleus accumbens and ventral pallidum (REF Jurek 2018). Integration of these animal studies with human neuroimaging (both ASD versus NT comparison studies, and intranasal intervention studies) starts offering an emerging picture of OT functioning in the human brain. At the level of the amygdala and prefrontal cortex, OT has been suggested to enhance social functioning by enhancing the salience of socially relevant information, such as eye gaze and facial and vocal emotional information [49,50]. Moreover, the anxiolytic effect of OT is postulated to be caused by high excitability of the oxytocinergic neurons in the lateral part of the central amygdala, which results in an inhibition of the motor fear response in the medial part of the central amygdala (REF Jurek 2018). In the reward system of the brain, OT acts as a social reinforcement signal and studies have shown its impact on perceived attractiveness of others (REF Scheele 2013; Striepens 2014).

The underlying cause of reduced endogenous OT levels in ASD children is still a topic of active research, but genetic differences have been put forward, possibly in interaction with environmental influences. OT expression and function are directly related to the OT and OT receptor genes, which in turn have been linked to autism symptomatology (REF Yrigollen 2008). For instance, certain haplotypes in the OT receptor gene confer risk for ASD (REF Lerer 2008). In addition, estrogen is known to regulate the transcription of the OT receptor gene in some brain regions, which might possibly explain some of the observed male-female disparities of the oxytocinergic system (REF Vaidyanathan et al., 2017). Besides genetics, the oxytocinergic system is also impacted by various (early-life) environmental factors, such as preterm birth, social environment, illness, trauma or stress (REF Buisman-Pijlman et al., 2014). The latter, for instance, has been shown to result in lower OT levels in adult men (REF Opacka-Juffry et al., 2011). Furthermore, during adulthood, the OT system is dynamic, especially during parturition and lactation where OT levels increase. Lastly, exposure to particular drugs, such as MDMA and methamphetamines, has also been shown to induce a strong OT release (REF Dumont et al., 2009).

The current study has some limitations that need to be taken into consideration. Regarding the methodology of the included studies, there is a high variability in numerous aspects of the process that could attribute to dubious results. First, phenotypic characterization was variable across studies. This implies that possible differences in symptom severity of ASD subjects or potential impact of previous treatment were not accounted for, which could correlate with the degree of impairment in the underlying OT biology. Second, different analysis approaches were used to measure OT concentration in samples (i.e., either ELISA or RIA techniques), which may also add to the heterogeneity of the findings [51]. Third, the included studies show a high variability in terms of age, race, distribution of sex within ASD and control samples and means of OT sampling measurement. While we incorporated the most prominent sources of variation in our analyses (i.e., age, sex and sample tissue), tissue extraction and analysis approach were not accounted for, which are known to yield large variability [52].

In conclusion, endogenous OT levels are lower in children with ASD as compared to NT controls, but this effect seems to disappear in adolescent and adult populations. Secondly, while no significant subgroup differences were found with regard to sex, the group difference in OT levels of individuals with versus without ASD seems to be only present in the studies with male participants. Finally, while no subgroup differences were found with regard to tissue sample, the ASD versus NT group difference in OT level may be slightly more pronounced for blood samples as compared to saliva samples. More research is required to investigate potential developmental changes in endogenous OT levels, both in typical and atypical populations. This research may also contribute to the design of more targeted therapies that can aid in mitigating differential OT development and its social consequences, e.g., through intranasal OT administration [17,53] or via interpersonal sensorimotor synchronization therapies that may aid in heightening OT’s endogenous production [54,55]. Further research employing more homogeneous methods is necessary to explore the possible use of OT level measurement as a diagnostic marker of ASD.

## Figures and Tables

**Table 1:** Overview of included study data, participant characteristics, and outcome.

Study	Subjects		Sample	Assay	[OT]		Unit	Results
	ASD	NT			ASD	NT		
OT levels in children (13)								
1. Abdulamir et al. (2016)	n = 60 (60 M) Age (y): 7.28 ± 2.89 DSM-5	n = 26 (26 M) Age (y): 6.92 ± 2.59	Plasma	ELISA	44.72 ± 36.1	102.1 ± 34.31	μIU/mL	Lower OT in ASD (*p* < 0.001)
2. Alabdali et al. (2014)	n = 50 (50 M) Age (y): 7.0 ± 2.34 DSM-IV; CARS, SRS	n = 30 (30 M) Age (y): 7.2 ± 2.14	Plasma	ELISA	71.71 ± 18.09	139.22 ± 36.62	μIU/mL	Lower OT in ASD (*p* = 0.001)
3. Feldman et al. (2014)	n = 40 (34 M/6 F) Age (m): 63.38 ± 12.35 DSM-5; ADOS-2	n = 40 Age (m): 53.56 ± 13.83	Saliva	ELISA	4.25 ± 0.66	6.89 ± 1.03	pg/mL	Lower OT in ASD (*p* < 0.05)
4. Fujisawa et al. (2014)	n = 15 (12 M/3 F) Age (m): 57.9 ± 13.6 DSM-5; DQ, PARS, SDQ	n = 58 (27 M/31 F) Age (m): 48.1 ± 22.7	Saliva	ELISA	39.33 ± 23.52 M: 40.3 ± 23.52 F: 35.7 ± 20.11	44.5 ± 24.89 M: 45.7 ± 29.78 F: 43.4 ± 20.15	pg/mL	No significant differences between groups (*p* = 0.449)
5. Husarova et al. (2016)	n = 19 (19 M) Age (m): 56.7 ± 25.4 ICD-10; CARS, ADI	n = 44 (44 M) Age (m): 58.9 ± 23.0	Plasma	ELISA	124.10 ± 90.59	267.77 ± 212.37	pg/mL	Lower OT in ASD (*p* = 0.0004)
6. Jacobson et al. (2014)	n = 37 (25 M/12 F) Age (y): 4.73 ± 0.61 DSM-IV-TR; ADI-R, ADOS	n = 41 (24 M/17 F) Age (y): 4.85 ± 0.61	Plasma	ELISA	M: 24.41 ± 7.45 F: 23.04 ± 6.97	M: 18,58 + 6.98 F: 22.59 + 8.82	pg/mL	Higher OT in male ASD only (*p* = 0.022)
7. Lakatosova et al. (2015)	n = 104 (80 M/24 F) Age (y): 7 ± 5.5 DSM-IV	n = 128 (103 M/25 F) Age (y): 10.5 ± 7	Plasma	ELISA	M: 208.1 ± 238.63F: 282.9 ± 318.92	M: 281.7 ± 200.85F: 340.7 ± 340.70	pg/mL	Lower OT in male ASD only (M: *p* = 0.0248; F: *p* = 0.5058)
8. Mariscal et al. (2019)	n = 34 (28 M/6 F) Age (y): 9.26 ± 0.37 DSM-IV-TR/DSM-V; ADI-R, ADOS	n = 30 (21 M/9 F) Age (y): 8.80 ± 0.40	Plasma	ELISA	8.62 ± 5.36	10.54 ± 5.37	pg/mL	No significant differences between groups (*p* = 0.1564)
9. Modahl et al. (1998)	n = 29 (29 M) Age (y): 8.1 + 1.7 DSM-IV	n = 30 (30 M) Age (y): 8.8 + 1.8	Plasma	RIA	0.64 ± 0.58	1.16 ± 0.77	pg/mL	Lower OT in ASD (*p* < 0.004)
10. Tanaka et al. (2020)	n = 12 (11 M/1 F) Age (m): 135 ± 16.7 DSM-IV-TR, DSM-V; CARS, ADOS, DISCO	n = 8 (4 M/4 F) Age (m): 107 ± 6.9	Saliva	ELISA	167.9 ± 62.01	161.5 ± 54.87	pg/mL	No significant difference between groups
11. Taurines et al. (2014)	n = 19 (19 M) Age (y): 10.7 ± 3.8 ICD-10; ADI-R, ADOS	n = 17 (17 M) Age (y): 13.6 ± 2.1	Plasma	RIA	19.6 ± 7.1	14.4 ± 9.6	pg/mL	No significant difference between groups (*p* = 0.132)
12. Yang et al. (2015)	n = 43 (35 M/8 F) Age (y): 7.51 ± 1.47 DSM-5; CARS	n = 40 (30 M/10 F) Age (y): 7.83 ± 1.63	Plasma	ELISA	116.47 ± 41.57	141.05 ± 51.61	pg/mL	Lower OT in ASD (*p* = 0.022)
13. Zhang et al. (2016)	n = 84 (71 M/13 F) Age (y): 3.95 ± 1.26 DSM-IV-TR; CARS	n = 85 (71 M/14 F) Age (y): 4.80 ± 1.22	Plasma	ELISA	20.05 ± 13.88	25.76 ± 15.30	pg/mL	Lower OT in ASD (*p* = 0.028)
**OT levels in adolescents (2)**								
14. Bakker-Huvenaars et al. (2020)	n = 49 (49 M) Age (y): 15.0 ± 2.1 DSM-5; DISC-IV	n = 28 (28 M) Age (y): 15.9 ± 1.8	Saliva	RIA	−0.22 ± 0.89	0.49 ± 0.97	z-score	Lower OT in ASD (*p* = 0.002)
15. Miller et al. (2013)	n = 40 (21 M/19 F) Age (y): M: 12.24 ± 3.56; F: 11.79 ± 3.43 DSM-IV-TR; ADOS	n = 35 (19 M/16 F) Age (y): M: 11.74 ± 2.49; F: 12.94 ± 3.19	Plasma	ELISA	M: 357.12 ± 126.05F: 525.23 ± 325.75	M: 361.52 ± 315.26F: 434.33 ± 332.27	pg/mL	No significant differences between groups (*p* = 0.270)
**OT levels in adults (3)**								
16. Althaus et al. (2016)	n = 31 (31 M) Age (y): 22.67 ± 4.22 DSM-IV-TR; ADOS,	n = 30 (30 M) Age (y): 22.67 ± 4.22	Plasma	RIA	1.34 ± 1.05	0.67 ± 0.77	pmol/L	Higher OT in ASD (*p* = 0.006)
17. Fujioka et al. (2020)	n = 17 (17 M) Age (y): 27.4 ± 7.2 DSM-IV; DISCO	n = 24 (24 M) Age (y): 29.0 ± 9.8	Saliva	ELISA	36.2 ± 13.2	43.6 ± 17.0	pg/mL	No significant difference between groups (*p* = 0.154)
18. Procyshyn et al. (2020)	n = 16 (16 F) Age (y): 29.9 ± 8.4 DSM-IV	n = 29 (29 F) Age (y): 27.2 ± 8.1	Saliva	ELISA	3.1 ± 0.5	2.8 ± 0.6	pg/mL	No significant difference between groups (*p* = 0.064)

OT = oxytocin (mean ± SD), ASD = autism spectrum disorder, NT = neurotypical, M = male, F = female, y = years, m = months, ELISA = enzyme-linked immunosorbent assay, RIA = radioimmunoassay. DSM-IV or DSM-5 Diagnostic and Statistical Manual of Mental Disorders Version IV or 5. ICD-10 = International Classification of Diseases Version 10. When applicable: confirmation of the ASD diagnosis using ADOS = Autism Diagnostic Observation Schedule or ADI(-R) = Autism Diagnostic Interview (-Revised) or CARS = Childhood Autism Rating Scale or DISCO = Diagnostic Interview for Social and Communication Disorders or DISC-IV Diagnostic Interview Schedule for Children Version IV3.2.

**Table 2:** The risk of bias evaluation for each individual study.

Study	Risk of Bias				Applicability Concerns		
	Patient Selection	Index Test	Reference Test	Flow and Timing	Patient Selection	Index Test	Reference Test
1. Abdulamir et al. (2016) [4]	?	?	☹	☺	☺	☺	☺
2. Alabdali et al. (2014) [5]	☹	?	☺	☹	☺	☺	☺
3. Feldman et al. (2014) [6]	?	?	☺	☹	☺	☺	☺
4. Fujisawa et al., 2014) [33]	?	?	☹	?	☺	☺	☺
5. Husarova et al. (2016) [9]	?	?	☺	?	☺	☺	☺
6. Jacobson et al. (2014) [34]	☹	?	☺	?	☺	☺	☺
7. Lakatosova et al. (2015) [35]	?	?	☹	☹	☺	☺	☺
8. Mariscal et al. (2019) [30]	☹	☺	☺	?	☺	☺	☺
9. Modahl et al. (1998) [7]	?	?	☹	?	☺	☺	☺
10. Tanaka et al. (2020) [31]	☹	?	☺	☹	☺	☺	☺
11. Taurines et al., 2014) [32]	?	?	☺	?	?	☺	☺
12. Yang et al. (2015) [10]	☹	?	☺	☹	☺	☺	☺
13. Zhang et al. (2016) [3]	?	?	?	?	☺	☺	?
14. Bakker-Huvenaars et al. (2016) [8]	☹	?	☺	☺	☺	☺	☺
15. Miller et al. (2013) [36]	☹	?	☺	?	☺	☺	☺
16. Althaus et al. (2016) [37]	?	?	☺	?	☺	☺	☺
17. Fujioka et al. (2020) [38]	☹	?	☺	?	☺	☺	☺
18. Procyshyn et al. (2020) [39]	?	?	☹	?	☺	☺	?

☺ = low risk, ☹ = high risk, ? = unclear risk.

## Supplementary Materials

The following are available online at https://www.mdpi.com/article/10.3390/brainsci11111545/s1, Figure S1: Subgroup analysis by age and sex.
