# Supplementary material for: Endogenous Oxytocin Levels in Autism—A Meta-Analysis
Source: Brain Sci. 2021 Nov 21;11(11):1545. doi: 10.3390/brainsci11111545 (PMC8615844; doi:10.3390/brainsci11111545)
Supplement: Supplementary file 1 [file brainsci-11-01545-s001.zip › brainsci-1456313-supplementary.pdf]

Supplementary

A two-factor subgroup interaction of age and sex revealed (marginally) lower OT levels in boys (n =595; g = -0.60; Z = 1.75; p = 0.08; CI = [-1.27, 0.07]) and mixed boys/girls (n = 416; g = -0.84; Z = 1.93; p = 0.05; CI = [-1.69, 0.01]) groups of children with ASD but not in girls with ASD (n = 112; g = -0.13; Z = 0.63; p = 0.53; CI = [-0.55, 0.28]) ((Figure S1).

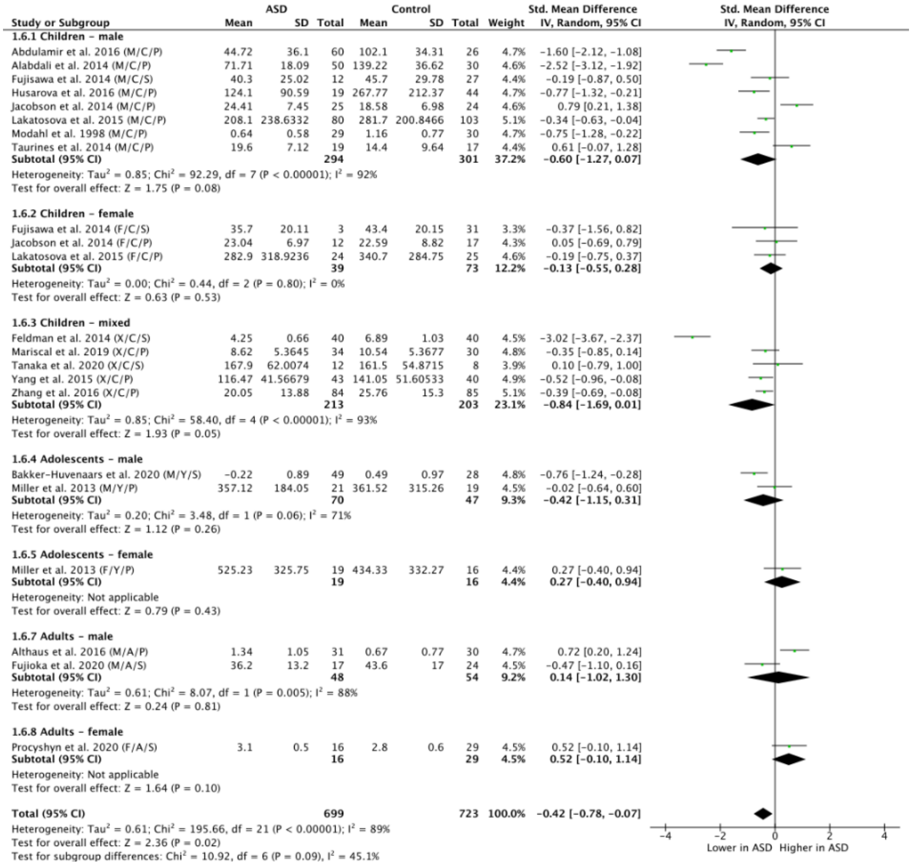

Figure S1. Subgroup analysis by age and sex.

M = male, F = female, X = mixed sexes; C = children, Y = youths/adolescents, A = adults; P = plasma, S = saliva.
